# Food and bile micelle binding of zwitterionic antihistamine drugs

**DOI:** 10.5599/admet.2454

**Published:** 2024-08-29

**Authors:** Rie Takeuchi, Kiyohiko Sugano

**Affiliations:** Molecular Pharmaceutics Lab., College of Pharmaceutical Sciences, Ritsumeikan University, 1-1-1, Noji-higashi, Kusatsu, Shiga 525-8577, Japan

**Keywords:** Negative food effect, unbound fraction, simulated intestinal fluid, dynamic dialysis, intestinal membrane permeation, zwitterionic, antihistamine

## Abstract

**Background and purpose:**

The food effects on oral drug absorption are challenging to predict from in vitro data. Food intake has been reported to reduce the oral absorption of several zwitterionic antihistamine drugs. However, the mechanism for this negative food effect has not been clear. The purpose of the present study was to evaluate the bile micelle and food binding of zwitterionic antihistamine drugs as a possible mechanism for the negative food effects on their oral drug absorption.

**Experimental approach:**

Bilastine (BIL), cetirizine (CET), fexofenadine (FEX), and olopatadine (OLO) were employed as model drugs. The fed/fasted AUC ratios of BIL, CET, FEX, and OLO after oral administration are reported to be 0.60 to 0.7, 0.92, 0.76 to 0.85, and 0.84, respectively. The unbound fraction (*f*_u_) of these drugs in the fasted and fed state simulated intestinal fluids (FaSSIF and FeSSIF, containing 3 and 15 mM taurocholic acid, respectively) with or without FDA breakfast homogenate (BFH) was measured by dynamic dialysis.

**Key results:**

The FeSSIF/ FaSSIF f_u_ ratios were 0.90 (BIL), 0.46 (CET), 0.76 (FEX), and 0.78 (OLO). In the presence of BFH, the f_u_ ratios were reduced to 0.52 (BIL), 0.22 (CET), 0.39 (FEX), and 0.44 (OLO).

**Conclusion:**

Despite being zwitterion at pH 6.5, the antihistamine drugs were bound to bile micelles. Bile micelle and food binding were suggested to cause a negative food effect on the oral absorption of these drugs. However, the AUC ratio was not quantitatively predicted by using FeSSIF + BFH.

## Introduction

The oral absorption of a drug is affected by various physiological factors in the gastrointestinal tract [1]. The physiological conditions in the fed state are markedly different from those in the fasted state. For example, bile micelle concentration is significantly increased in the fed state. In addition, food components can interact with drug molecules. It is still challenging to predict the food effects on oral drug absorption from in vitro data [2-4]. In the mechanistic oral absorption models, the free (unbound) fraction in the intestinal fluid (*f*_u_) is one of the key parameters that determine the effective intestinal permeability of a drug (*P*_eff_) [5]. High solubility/ low permeability drugs tend to show a negative food effect. In these cases, the rate and extent of fraction dose absorbed *F*_a_ is limited by epithelial membrane permeation (*F*_a_ rate-limiting step (FaRLS): permeability-limited (PL) by the epithelial membrane (PL-E)) [2,6]. Recently, the bile micelle and food binding of drugs were reported to be able to elucidate the negative food effect for hydrophilic tertiary and quaternary amines [5,7]. Some zwitterionic antihistamine drugs are also known to show negative food effects in humans. However, it has not been clear whether a zwitterionic drug can also bind to bile micelles and/or food components.

**Figure 1.**
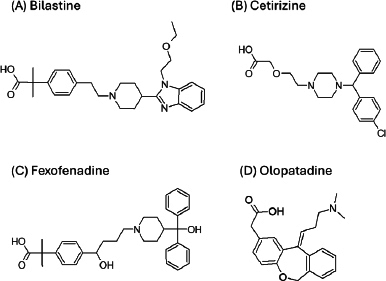
Chemical structures of zwitterionic antihistamine model drugs

The purpose of the present study was to investigate whether zwitterionic antihistamine drugs can bind to bile micelles and food components. Bilastine (BIL), cetirizine (CET), fexofenadine (FEX), and olopatadine (OLO) were employed as model drugs (Figure 1). The physicochemical properties of these drugs are shown in Table 1. These drugs are zwitterionic and show moderate lipophilicity at pH 6.5. The fed/fasted AUC ratios of BIL, CET, FEX, and OLO are 0.60 to 0.7 [8-10], 0.92 [11], 0.76 to 0.85 [12,13], and 0.84 [14], respectively. In this study, dynamic dialysis was used to measure the *f*_u_ values in the simulated intestinal fluids consisting of bile micelles and food homogenates.

**Table 1.** Physicochemical properties and clinical data of model drugs

Drug	MW	p*K*_a_^a^	Isoelectric point	log *D*_oct_ pH 6.5^b^	Dose, mg	Food effect, %^c^
Bilastine	464	4.0 (A) 8.8 (B)	6.40	0.39 ± 0.01	20	0.60 – 0.70
Cetirizine	389	2.12 (B) 2.90 (A) 7.98 (B)	5.44	1.46 ± 0.01	10	0.92
Fexofenadine	502	4.20 (A) 7.84 (B)	6.02	0.51 ± 0.01	120	0.76 – 0.85
Olopatadine	337	4.18 (A) 9.79 (B)	6.99	0.34 ± 0.01	10	0.84

^a^ Bilastine (estimated from the pH - log *D*_oct_ profile. Temperature and ionic strength not reported) [8], cetirizine (25 ˚C, *I* = 0.15 M) [15], fexofenadine (24 ˚C, *I* = 0.01 M) [15], and olopatadine (Temperature and ionic strength not reported) [14].

^b^ Measured in this study

^c^ AUC ratio (fed/fasted). Bilastine [8], cetirizine [11], fexofenadine [12] and olopatadine [14].

The permeation flux (*J*) is expressed by the total drug concentration dissolved in the GI fluid (*C*_dissolv_ = unbound + bound) and the effective (apparent) permeation coefficient (*P*_eff_) as in Equation (1) [2]:



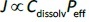

(1)


In the case of PL-E, according to the free fraction theory, only unbound molecules can permeate the epithelial membrane, Equation (2),



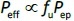

(2)


where *P*_ep_ is the epithelial membrane permeation coefficient defined based on the unbound drug concentration, and *f*_u_ is the unbound (free) fraction. Bile micelle and food binding can reduce the concentration of unbound drug molecules (= *f*_u_ *C*_dissolv_) at the epithelial membrane surface. In the case when the oral absorption of a drug is permeability-limited, *F*_a_ can be calculated as in Equation (3)



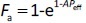

(3)


where *A* = 1.4 × 10^4^ s/cm (*P*_eff_ in cm/s). The *F*_a_ ratio (= AUC ratio) in the fasted/fed states approximately becomes the *f*_u_ ratio (*f*_u_*,_fed_*/*f*_u_,_fasted_) for *F*_a_ < 0.7 cases [2].

## Experimental

### Material

Fexofenadine hydrochloride, bilastine, olopatadine hydrochloride, and cetirizine dihydrochloride were purchased from Tokyo Chemical Industry Co., Ltd (Tokyo, Japan). Sodium chloride (NaCl), sodium dihydrogen phosphate dihydrate (NaH_2_PO_4_ 2H_2_O), 8N NaOH, 1-octanol, taurocholic acid (TC), and oleic acid (OA) were purchased from FUJIFILM Wako Pure Chemical Corporation (Osaka, Japan). Egg yolk lecithin (EL) was purchased from Kewpie Corporation (Tokyo, Japan). Glyceryl mono-oleate (GM) was purchased from Nippon Surfactant Industries Co., Ltd (Tokyo, Japan). A cellulose dialysis membrane (*Φ* 15.9×25 mm×15 m) was purchased from As-One Corporation (Osaka, Japan).

The FDA breakfast ingredients, and other food were purchased from the local market (bacon (Itoham Foods Inc, Japan), toast (Choujuku bread, Pasco Shikishima Corporation, Japan), egg (Akadama, miwa keien Co., Ltd, Japan), hash browns (Hoshino potato, Heinz Japan Ltd, Japan), whole milk (Oishii Megumilk Snow Brand Milk, MEGMILK SNOW BRAND Co., Ltd, Japan), butter (Hokkaido-butter, MEGMILK SNOW BRAND Co., Ltd, Japan).

### Methods

#### Processing of FDA Breakfast

The FDA breakfast was comprised of one strip of bacon, half a slice of toast, one fried egg, 55 g of hash browns, 30 g of butter, and 100 mL of whole milk. After cooking, the FDA breakfast was homogenized for 15 s by a food processor. The FDA breakfast homogenate (BFH) was divided into small aliquots and stored in a freezer (-30 °C). It was thawed under running water before use.

#### Measurement of the unbound fraction by dynamic dialysis

Dynamic dialysis was performed using a side-by-side chamber (SANPLATEC Co., Ltd (Osaka, Japan)) with a cellulose dialysis membrane (molecular weight cut-off: 3500). The membrane area was 2.0 cm^2^. The fluid volume was 1.5 mL for both the donor and acceptor sides. The fasted and fed state simulated intestinal fluids (FaSSIF and FeSSIF, respectively) with or without BFH were used as test media (Table 2). Each drug was dissolved in the test media and added to the donor side (0.04 mM (BIL), 0.2 mM (CTZ), 0.5 mM (FEX), and 0.5 mM (OLO)). The blank FaSSIF was added to the acceptor side. After incubation for 1 h at 37 °C, the drug concentration in the acceptor side was measured by HPLC (Shimazu Prominence LC-20 series and Agilent Technologies 1200 Series, column: ZORBAX Eclipse Plus (C18 2.1×50 mm, 3.5 μm) (Agilent Technologies), flow rate: 0.6 mL min^-1^, mobile phase: 0.1 % trifluoroacetic acid-acetonitrile/0.1 % trifluoroacetic acid-water (25 % (BIL), 35 % (CTZ), 35 % (FEX), and 28 % (OLO)), detection: UV (BIL: 280 nm, CET: 230 nm, FEX: 225 nm, and OLO: 300 nm), column temperature: 40 °C, and injection volume of 10 μL).

**Table 2.** Composition of simulated intestinal fluids ^a^

Test medium	Content, mM				BFH, %^c^
	TC	EL	GM	OA	
FaSSIF	3	0.75	0	0	0
FeSSIF	15	3.75	0	0	0
×2FeSSIF	30	7.50	0	0	0
FeSSIFv2	10	2.0	5	0.8	0
BFH ^b^	0	0	0	0	50
FeSSIF+BFH	15	3.75	0	0	50

^a^ The components were dissolved in a phosphate buffer (phosphate 28.6 mM, NaCl 106 mM, pH 6.5) (blank FaSSIF). TC: taurocholic acid, EL: egg lecithin, GM: Glyceryl mono-oleate, OA: oleic acid.

^b^ BFH added to blank FaSSIF.

^c^ wt.%

Permeation was calculated as the ratio of the concentration in the acceptor side at 1 h and the theoretical equilibrium concentration (0.02 mM (BIL), 0.1 mM (CTZ), 0.25 mM (FEX), and 0.25 mM (OLO)) (×100 to convert to %). The unbound fraction (*f*_u_) was calculated as the ratio of permeation in each medium and the test medium of Blank FaSSIF.

#### The octanol-water distribution coefficient measurement

The octanol-water distribution coefficient (log *D_oct_*) values of model drugs were measured by the shake-flask method. A model drug was dissolved in blank FaSSIF pre-saturated with 1-octanol (BIL: 0.1 mM, CTZ: 1 mM, FEX: 1 mM, and OLO: 1 mM). The drug solution (2.5 mL or 5.5 mL) was added to 0.5 mL of 1-octanol pre-saturated with water. The samples were shaken by a shaker for 90 minutes at room temperature (25 ± 2 °C). The drug concentration in the aqueous phase was measured by a UV absorbance (BIL: 280 nm, CET: 230 nm, FEX: 225 nm, and OLO: 300 nm, UV-1850, Shimadzu Corporation, Kyoto, Japan). The log *D*_oct_ value was calculated by Equation 4:



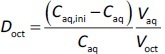

(4)


where *C*_aq,ini_ and *C*_aq_ are the initial and equilibrium drug concentrations in the aqueous phase, and *V*_aq_ and *V*_oct_ are the volume of aqueous and octanol phases, respectively.

## Results and discussion

**Figure 2.**
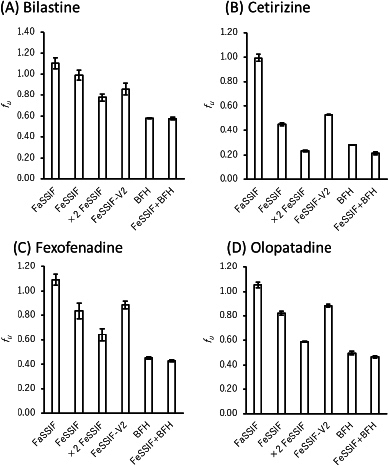
Effect of bile micelles and FDA breakfast homogenate (BFH) on the *f*_u_ values of zwitterionic antihistamine drugs.

The *f*_u_ values of all drugs decreased in the presence of bile micelles and BFH (Figure 2, Table 3). This result suggests that the reduction of *f*_u_ by bile micelle binding in the fed state is likely to be one of the reasons for the negative food effect on the oral absorption of these drugs. The addition of BFH also decreased the *f*_u_ values of model drugs. Therefore, direct food binding could also be one of the reasons for the negative food effect.

**Table 3.** Permeation and *f*_u_^a^

Drug	Test medium	Permeation at 1 h, %	*f*_u_	*f*_u_ *ratio ^b^*
Bilastine	Blank FaSSIF	15.6 ± 0.5		
	FaSSIF	17.3 ± 0.8	1.10 ± 0.05	
	FeSSIF	15.5 ± 0.7	0.99 ± 0.05	0.90 ± 0.04
	×2 FeSSIF	12.1 ± 0.6	0.78 ± 0.04	0.70 ± 0.03
	FeSSIF-V2	13.4 ± 0.8	0.86 ± 0.06	0.78 ± 0.05
	BFH	9.0 ± 0.1	0.58 ± 0.00	0.52 ± 0.00
	FeSSIF+BFH	9.0 ± 0.1	0.58 ± 0.01	0.52 ± 0.01
Cetirizine	Blank FaSSIF	16.4 ± 0.6		
	FaSSIF	16.3 ± 0.5	0.99 ± 0.03	
	FeSSIF	7.4 ± 0.2	0.45 ± 0.01	0.46 ± 0.01
	×2 FeSSIF	3.8 ± 0.1	0.23 ± 0.01	0.23 ± 0.01
	FeSSIF-V2	8.7 ± 0.1	0.53 ± 0.00	0.53 ± 0.00
	BFH	4.6 ± 0.0	0.28 ± 0.00	0.29 ± 0.00
	FeSSIF+BFH	3.5 ± 0.2	0.21 ± 0.01	0.22 ± 0.01
Fexofenadine	Blank FaSSIF	12.0 ± 0.7		
	FaSSIF	13.1 ± 0.5	1.09 ± 0.04	
	FeSSIF	10.0 ± 0.7	0.84 ± 0.06	0.76 ± 0.06
	×2 FeSSIF	7.7 ± 0.6	0.64 ± 0.05	0.59 ± 0.05
	FeSSIF-V2	10.6 ± 0.4	0.88 ± 0.03	0.81 ± 0.03
	BFH	5.4 ± 0.1	0.45 ± 0.01	0.41 ± 0.01
	FeSSIF+BFH	5.1 ± 0.1	0.43 ± 0.01	0.39 ± 0.01
Olopatadine	Blank FaSSIF	20.8 ± 0.7		
	FaSSIF	21.9 ± 0.5	1.05 ± 0.02	
	FeSSIF	17.1 ± 0.3	0.82 ± 0.02	0.78 ± 0.02
	×2 FeSSIF	12.2 ± 0.1	0.59 ± 0.01	0.56 ± 0.01
	FeSSIF-V2	18.4 ± 0.3	0.88 ± 0.01	0.84 ± 0.01
	BFH	10.3 ± 0.3	0.50 ± 0.02	0.47 ± 0.01
	FeSSIF+BFH	9.7 ± 0.2	0.46 ± 0.01	0.44 ± 0.01

^a^Mean ± S.D., *n* = 3

^b^*vs.* the *f_u_* value in FaSSIF

The *f*_u_ ratios of FeSSIF/FaSSIF of FEX and OLO were in good agreement with the extent of the food effect in humans. However, the food effect was underestimated for BIL and overestimated for CET. In the presence of BFH, the *f*_u_ ratio of FeSSIF/FaSSIF of BIL was in good agreement with the food effect. Even though the *f*_u_ ratio of CET is lower than that of the other drugs, the clinical negative food effect on the oral absorption of CET is less significant. This would be due to the higher lipophilicity and faster passive permeability of CET. In such case, the extent of *F*_a_ becomes less sensitive to a decrease in *f*_u_ and *P*_eff_ (Eq. 3). The bioavailability in humans of CET is > 70 % [11], which is higher than that of FEX (33 %) [16] and BIL (61 %) [8].

**Figure 3.**
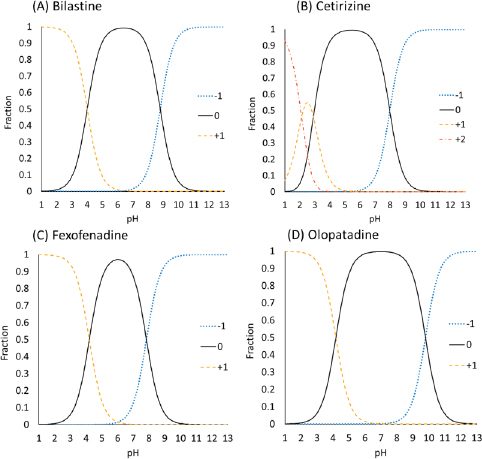
Charge species distribution of zwitterionic antihistamine drugs

The charge species distribution of the model drugs is shown in Figure 3. Despite being zwitterion at pH 6.5, the antihistamine drugs were bound to bile micelles. The bile micelle binding of CET was stronger than that of others, as expected from its higher log *D*_oct_ (CET > FEX ≈ BIL ≈ OLO). CET is a triprotic compound [17,18]. However, the first p*K*_a_ (N1 of the piperidine group) is 2.12 and is not protonated at pH 6.5. Therefore, CET mainly exists as a zwitterion at pH 6.5.

The log *D*_oct_ values of BIL (0.39) and OLO (0.34) are close to that of propranolol (PRO) (log *D*_oct_ = 0.4) [5]. In addition, the amine groups of these drugs are > 99 % charged at pH 6.5. However, the bile micelle binding of propranolol is reported to be about 3-fold stronger than those of BIL and OLO. In addition, even though the log *D*_oct_ values of the zwitterions are greater than that of quaternary ammonium compounds (QAC) (log *D*_oct_ ≪ 0), the *f*_u_ values of the zwitterionic drugs are comparable with that of QACs [7]. The bile micelles in FaSSIF and FeSSIF are negatively charged by the -SO_3_^-^ group in taurocholic acid. Therefore, the carboxyl anion (-COO^-^) in the zwitterionic drugs was suggested to reduce the bile micelle binding, like in the cases of liposome binding [18,19]. In a buffer solution at the isoelectric pH point, a zwitterion drug exists as an equilibrium between a zwitterion molecule and an un-ionized molecule [20]. The isoelectric pH points of the model drugs are near pH 6.5 (5.4 to 7.0) (Table 1). From the difference between the p*K*_a_ values of carboxylic acid and amine (> 3.6), they are estimated to exist as the zwitterion more than 99.8 % [21]. Therefore, the zwitterionic molecules may bind bile micelles. A more detailed mechanism of the charge effect on the bile micelle binding of drugs is under investigation.

## Conclusions

In conclusion, the zwitterionic antihistamine drugs were found to bind to bile micelles and foods, suggesting that it would be the reason for the negative food effect. The lipophilicity and charge state were suggested to affect the bile micelle binding. Dynamic dialysis would be a good tool to measure bile micelle and food binding.
